# Crosstalk between Long Non-Coding RNA and Spliceosomal microRNA as a Novel Biomarker for Cancer

**DOI:** 10.3390/ncrna9040042

**Published:** 2023-07-31

**Authors:** Maram Arafat, Ruth Sperling

**Affiliations:** Department of Genetics, The Hebrew University of Jerusalem, Jerusalem 91904, Israel

**Keywords:** lncRNA, miRNA, spliceosomal miRNA, cancer, supraspliceosome

## Abstract

Non-coding RNAs (ncRNAs) play diverse roles in regulating cellular processes and have been implicated in pathological conditions, including cancer, where interactions between ncRNAs play a role. Relevant here are (i) microRNAs (miRNAs), mainly known as negative regulators of gene expression in the cytoplasm. However, identification of miRNAs in the nucleus suggested novel nuclear functions, and (ii) long non-coding RNA (lncRNA) regulates gene expression at multiple levels. The recent findings of miRNA in supraspliceosomes of human breast and cervical cancer cells revealed new candidates of lncRNA targets. Here, we highlight potential cases of crosstalk between lncRNA and supraspliceosomal miRNA expressed from the same genomic region, having complementary sequences. Through RNA:RNA base pairing, changes in the level of one partner (either miRNA or lncRNA), as occur in cancer, could affect the level of the other, which might be involved in breast and cervical cancer. An example is spliceosomal mir-7704 as a negative regulator of the oncogenic lncRNA HAGLR. Because the expression of spliceosomal miRNA is cell-type-specific, the list of cis-interacting lncRNA:spliceosomal miRNA presented here is likely just the tip of the iceberg, and such interactions are likely relevant to additional cancers. We thus highlight the potential of lncRNA:spliceosomal miRNA interactions as novel targets for cancer diagnosis and therapies.

## 1. Introduction

Cancer is a disease that modifies cellular homeostasis and promotes uncontrolled cell growth. Cancer can be caused by genetic mutations, epigenetic alterations, chromosomal translocations, deletions, and splicing mis-regulation that affects protein-coding genes [1,2,3]. In addition, non-coding RNAs (ncRNAs), transcripts that are encoded by the genome but are mostly not translated into proteins, appear to play major roles in cancer [1,4,5].

A subgroup of ncRNA is the small non-coding RNA (sncRNA), a group of essential regulatory factors in splicing and gene expression, which also plays a role in cancer and part of them are expressed from introns. Among the members of sncRNA are microRNA (miRNA), mainly known for its role in inhibiting translation in the cytoplasm and small nucleolar RNA, known for its role in modifying non-coding RNA in the nucleolus [4]. Another subgroup of ncRNA is the group of long ncRNA (lncRNA), known to be involved in the regulation of chromatin dynamics, transcriptional regulation of coding genes, maintenance of genomic integrity, X-chromosome inactivation, genomic imprinting, cell differentiation, and development [1,6,7].

Splicing and alternative splicing play a major role in the diversity of the proteome. Furthermore, aberrations in splicing are found in cancer [8,9,10,11]. Splicing and alternative splicing [8,9,10,11,12] take place in the endogenous spliceosome—the supraspliceosome. This is a huge (21 MDa), highly dynamic machine comprising four active native spliceosomes joined together by the pre-mRNA. The supraspliceosome is an autonomous macromolecular machine, where all nuclear pre-mRNAs, regardless of their length or number of introns, are individually assembled and processed in a carefully controlled, coordinated fashion. The tetrameric structure of the supraspliceosome is suitable to coordinate multiple processing events of the pre-mRNA, including alternative splicing [4,13,14].

ncRNAs are found in the spliceosome and, through RNA:RNA base pairing, play a major role in the regulation of gene expression, including splicing. The five spliceosomal U snRNAs play a major role in splicing accuracy and catalysis through dynamic changes in U snRNA:pre-mRNA and U snRNA:U snRNA base-pairing interactions [8,9,10,11,12]. The identification of additional ncRNA within the supraspliceosome raises the potential for novel functional RNA:RNA base-pairing interactions [4]. Because a large number of miRNA and small nucleolar RNA are embedded in introns, it was hypothesized that their biogenesis might occur within the endogenous spliceosome [15]. DGCR8 and DROSHA, the main microprocessor components, were found within the supraspliceosome, and a crosstalk between splicing and the biogenesis of intronic miRNA was demonstrated [15]. Furthermore, sequencing of small RNA within the supraspliceosome revealed a large collection of sncRNA [5,15,16,17,18] including miRNA, and among these, miRNA not embedded in introns [5,18], and SNORD sequences [17]. The finding of non-intronic miRNA within the supraspliceosome suggested that their presence there signifies novel functions within the spliceosome [5,18] (see below). Furthermore, spliceosomal SNORD27, lacking the methylase fibrillarin, was demonstrated to regulate alternative splicing of the transcription factor E2F7 through base pairing [17]. These studies indicate that spliceosomal sncRNA assembled in non-canonical complexes and through different base pairing than their canonical ones can potentially target novel and yet unexplored RNA targets [4,5,18].

Here, we highlight a new aspect of lncRNAs in cancer, acting through cis-crosstalk (close interaction), through base pairing with miRNA present within the endogenous spliceosome when both are expressed from the same genomic region. 

## 2. ncRNA in Cancer

### 2.1. MicroRNA (miRNA)

miRNAs are small molecules ~22 nt in length, which play an essential role in regulating cellular signaling pathways, and changes in their expression level are associated with several diseases including cancer. Their main known role is in the suppression of gene expression and translation, mostly by base pairing to the 3’UTR region of target mRNA transcripts in the cytoplasm [4,19]. However, the finding of miRNA in the nucleus raised the options for additional functions for miRNA in the nucleus [20,21,22]. Although the functions of miRNAs in the nucleus are not yet well understood, recent reports demonstrated their involvement in several processes, such as the regulation of ncRNA, transcriptional silencing, transcription inhibition, and transcription activation [23,24,25,26]. 

Recently, the link between miRNAs and various cancers or other diseases has been widely researched. These studies lead to numerous miRNA-based potential cancer biomarkers for cancer diagnosis and prognosis which were put forward, providing a new perspective on cancer screening [27].

### 2.2. miRNA in the Supraspliceosome

Recent studies demonstrated the presence of mature miRNAs and pre-miRNA-derived sequences within the supraspliceosome, suggesting novel nuclear functions, in addition to their known cytoplasmic ones [4,5,18]. 

A recent study revealed differences in the expression levels of miRNA in the spliceosome fractions compared to cytoplasmic miRNA in HeLa cells [18]. A further study of breast-derived cell lines with increased tumorigenicity (from MCF-10A, non-malignant, to malignant MCF-7 and metastatic MDA-MB-231) revealed cell-line-specific changes in the spliceosome fractions with malignancy, including changes in expression level, identity, and pre-miRNA segmental composition [5]. Notably, the changes in expression of the majority of the abundant spliceosomal miRNA show an opposite trend in view of the literature and breast cancer large cohorts [5]. The results suggest that spliceosomal miRNAs act in the nucleus on alternative targets than in the cytoplasm. An example is spliceosomal mir-7704 (discussed below) whose genomic location overlaps HOXD Antisense Growth-Associated Long Non-Coding RNA (HAGLR), a cancer-related lncRNA [5]. These findings suggest spliceosomal miRNAs as novel potential targets for therapeutic avenues for cancer.

### 2.3. Long Non-Coding RNA (lncRNA)

In mammals and other eukaryotes, part of the genome is regulated by numerous lncRNAs, considered being longer than 200 nt, transcribed by RNA polymerase II, but mainly not translated to proteins [7,28,29]. lncRNA can regulate gene expression epigenetically at transcriptional, post-transcriptional, translational, and post-translational levels. This regulation occurs via lncRNA interaction with DNA, RNA, ncRNA, proteins, and combinations of them, including trans interactions on targets distant from the lncRNA transcription site and cis interactions on targets close to the lncRNA transcription site [30]. Several cases of cis-acting lncRNA, which activate or repress other genes via interaction with the DNA of promoters or enhancers when complexed with proteins, have been studied [31]. Although lncRNA is considered a class of ncRNA, the large numbers of lncRNAs and their diversity lead to divide them into additional categories like genomic localization, function, and structure.

#### 2.3.1. Genomic Localization

Genomic localization includes intronic lncRNAs located in introns of the coding region; intergenic lncRNAs (lincRNA) derived from the region between two coding genes; enhancer lncRNAs (elncRNA) originated from promoter enhancer regions; bi-directional lncRNAs localized close to a coding transcript of the opposite strand; sense-overlapping lncRNAs overlapped with introns and exons of different coding transcripts in the sense strand of the DNA; and antisense transcripts derived from the antisense strands of the DNA, which may or may not be complementary to coding sequences in the sense strand [1,6]. 

#### 2.3.2. Function

lncRNAs are classified as signaling—lncRNA expression can reflect the actions of transcription factors or signaling pathways to indicate gene regulation; decoy—lncRNAs can interact and keep away transcription factors and other proteins from their chromatin targets, thus facilitating gene activation or silencing; and guide—lncRNA can bind to protein complexes and guide them to a specific DNA target, thus regulating signaling events and gene expressions. Target genes can be either in cis, near the site of lncRNA production, or in trans, acting on distant target genes, and scaffold includes lncRNAs that can bring together multiple proteins to form ribonucleoprotein (RNP) complexes, which can regulate gene expression and chromosomal dynamics [1,7,30].

#### 2.3.3. Structure

lncRNAs with similar short motif elements, although missing general homology, have analogous functions, suggesting that repeat structures are essential elements of lncRNAs that could determine lncRNA function [7].

### 2.4. Competing Endogenous RNA (ceRNA) Networks and Regulation

ncRNAs play diverse roles in cellular processes and have been implicated in many pathological conditions, including cancer. Interactions between subclasses of ncRNA play a role in regulating gene expression and so does their interaction with coding transcripts. Several studies have demonstrated the involvement of these ncRNAs in competitive regulatory interactions, known as competing endogenous RNA (ceRNA) networks [32]. An example is the case of lncRNAs that act as microRNA decoys to modulate gene expression [33].

A notable impact of such a network is that the interactions are often interconnected. Thus, aberrant expression of any network component could affect the complex regulatory circuitry, culminating in cancer development and progression or other diseases [32,33].

### 2.5. lncRNA in Cancer

Numerous lncRNAs are aberrantly expressed in various tumors, and some appear to be cancer-specific. In addition, their expression levels can serve as a marker for the severity of the disease. All these data make lncRNAs attractive candidates as biomarkers and therapeutic targets for the treatment of cancer [1,34,35,36,37]. 

## 3. lncRNA: Spliceosomal miRNA Crosstalk in Breast and Cervical Cancer Cells

In this review, we want to highlight the potential impact on gene expression of the crosstalk between lncRNA and miRNA, which are expressed from the same genomic region, thus having complementary or identical sequences. Such a crosstalk is possible when the relevant miRNA is found within the supraspliceosome. Specifically, we will focus here on a selected number of lncRNAs, those that might be affected by miRNA expressed in the spliceosome of both breast and cervical cancer cells [5,18].

Analysis of miRNAs found in the supraspliceosome fractions of human breast and cervical cancer revealed several cases of spliceosomal miRNA expressed from the same genomic region, yet in the opposite direction to a specific lncRNA, highlighting these lncRNAs as antisense transcripts to these miRNAs (Table 1 and Table 2). The analysis of spliceosomal miRNA in breast cancer cells revealed 191 miRNAs expressed in the supraspliceosome of breast cancer cells [5]; among them, 20 have overlapping genetic locations with 18 lncRNAs, and 9 of these miRNAs are expressed in the antisense direction to 7 lncRNAs and thus can interact through RNA:RNA base pairing. Table 1 summarizes the changes in the expression of these latter 20 spliceosomal miRNAs in human breast cancer cells compared to normal human breast cells and the percentage of mature miRNA sequences in the respective spliceosomal miRNA [5]. It also indicates the overlapping lncRNA and the genomic annotation of the miRNA relative to the respective lncRNA. 

Analysis of the expression of spliceosomal miRNA in human cervical cancer cells and comparison with miRNA in the cytoplasm of these cells [18] revealed 200 miRNAs expressed in the supraspliceosome, 26 of which overlap with the genomic locations of 22 lncRNAs [18]. Notably, 15 of the spliceosomal miRNAs are expressed in the antisense direction to that of 12 lncRNAs (Table 2).

It should be pointed out that the miRNA sequences found within the supraspliceosome are not limited to mature miRNA, and sequences aligning to different regions of the pre-miRNA are also found there. Furthermore, the pre-miRNA segmental composition of spliceosomal miRNA is cell-type-specific [5,18]. Table 1 and Table 2 also include the percentage of mature miRNA for each listed spliceosomal miRNA. Notably, in several of the cases listed above, the spliceosomal miRNA is composed of mature miRNA. When the percentage of mature miRNA is less than 100%, the rest of the population is composed mainly of sequences of “overlap regions” of the pre-miRNA sequence [5,18]. Importantly, in the studied breast cancer cell lines (MCF-10A, compared to breast cancer cells MCF-7 and MDA-MB-231), the percentage of mature spliceosomal miRNA in the non-malignant MCF-10A cells is only 25% and increases to ~60% and 68% in the breast cancer MCF-7 and MDA-MB-231 cells, respectively [5].

**Table 1:** Spliceosomal miRNA sequences expressed from the same genomic location of lncRNA human breast cancer cells.

miRNA	Reads			miRNA Strand	lncRNA ^b^			lncRNA Strand	miRNA Annotation Relative to lncRNA
	MCF10A (% Mature) ^a^	MCF7 (% Mature)	MDA (% Mature)		Name	Genomic Coordinates ^c^	Accession No.		
**Antisense direction (lncRNA and miRNA)**									
* has-mir-7704	1167 (100)	80 (100)	94 (100)	+	HAGLR	chr2:176173189-176188958	NR_033979.2	−	exon
hsa-mir-99b	23 (95.65)	37 (94.59)	69 (100)	+	SPACA6P-AS1	chr19:51685363-51693456	NR_108100.1	−	Splice junction
hsa-let-7e	66 (19.7)	24 (91.67)	23 (100)	+					exon
hsa-mir-125a	25 (84)	10 (100)	16 (87.5)	+					exon
hsa-let-7i	76 (97.37)	138 (98.55)	661 (99.09)	+	LINC01465	chr12:62601751-62603690	ENST00000408887.3	−	Part of exon (first 5 nt)
hsa-mir-29a	27 (11.11)	0	39 (87.18)	−	LINC00513	chr7:130853732-130930680	ENST00000653887.1	+	intron
					LINC-PINT	chr7:130791264-131107928	ENST00000642963.1	−	intron/exon
hsa-mir-16-2	0	0	14 (100)	+	TRIM59-IFT80	Isoform 1: chr3:160227454-160449838	NR_148401.1	−	intron
						Isoform 2: chr3:160256986-160449838	NR_148402.1		
						Isoform 3: chr3:160256986-160485747	NR_148403.1		
* has-mir-1246	1814 (100)	189 (100)	144 (100)	−	LINC01117	chr2:176495255-176655967	ENST00000652995.1	+	intron
hsa-let-7d	0	0	24 (83.33)	+	LINC02603	Isoform 1: chr9:94176569-94204566	NR_046163.1	−	intron
						Isoform 2: chr9:94176570-94204566	NR_046165.1		
						Isoform 3: chr9:94176569-94259311	NR_160773.1		
**Sense direction (lncRNA and miRNA)**									
hsa-mir-92b	0	0	26 (100)	+	THBS3-AS1	Isoform 1: chr1:155194997-155205489	NR_183234.1	+	exon
						isoform 2: chr1:155194997-155205489	NR_183235.1		
						Isoform 3: chr1:155194997-155205489	NR_183236.1		
						Isoform 4: chr1:155194997-155205489	NR_183237.1		
						Isoform 5: chr1:155194997-155205489	NR_183238.1		
* has-mir-612	1494 (0.47)	0 (0)	20 (0)	+	NEAT1	chr11:65422798-65445540	NR_131012.1	+	exon
hsa-mir-30c-2	0	0	39 (100)	−	LINC00472	Isoform 1: chr6:71343427-71420130	ENST00000651778.1	−	intron/exon
* hsa-mir-30a	19 (100)	0	473 (97.67)	−		Isoform 2: chr6:71373251-71420148	ENST00000625013.2		intron
hsa-mir -4712	21 (90.48)	0	0	+	GABPB1-AS1	Isoform 1: chr15:50355476-50372158	ENST00000648591.1	+	intron/exon
						Isoform 2: chr15:50355467-50372202	ENST00000499624.3		
hsa-mir-16-1	0	0 (0)	13 (76.92)	−	DLEU2	Isoform 1: chr13:49982549-50125541	NR_152566.1	−	intron
						Isoform 2: chr13:50043436-50082041	NR_152567.1		
						Isoform 3: chr13:50043436-50082041	NR_152568.1		
						Isoform 4: chr13:50049189-50082041	NR_152569.1		
hsa-mir-15a	42 (0)	27 (3.7)	33 (3.03)	−		Isoform 5: chr13:50026695-50082041	NR_152571.1		intron/part of exon
						Isoform 6: chr13:50049189-50082041	NR_152570.1		
						Isoform 7: chr13:50026695-50082041	NR_152572.1		
hsa-mir-1204	22 (0)	0	0	+	PVT1	chr8:127794533-128101253	NR_003367.3	+	intron
hsa-mir-545	10 (0)	0	0	−	FTX	chrX:74028136-74293574	NR_028379.1	−	intron
hsa-mir-6516	10 (0)	0	0	+	SNHG20	chr17:77088643-77094986	NR_027058.1	+	intron
					SCARNA16	chr17:77089307-77089493	NR_003013.1		exon
hs-mir-6840	85 (0)	21 (0)	13 (0)	+	STAG3L5P-PVRIG2P-PILRB	Isoform 1: chr7:100336065-100367831	NR_036569.1	+	intron
						Isoform 2: chr7:100336065-100367831	NR_036570.1		

* Indicates one of the most abundant spliceosomal miRNAs in the above cells according to Mahlab-Aviv et al., 2020 [5]. ^a^ Percentage mature miRNA; MCF-10A (MCF10A), MCF-7 (MCF7), and MDA-MB-231 (MDA); ^b^ excluding lncRNA which are miRNA host genes; and ^c^ GRCh38/hg38. Table composed from data of ref. [5].

**Table 2:** Spliceosomal miRNA sequences expressed from the same genomic location of lncRNA HeLa cells.

miRNA	Reads		miRNA Strand	lncRNA ^d^			lncRNA Strand	miRNA Annotation Relative to lncRNA
	SF HeLa ^a^ (% Mature) ^c^	CE HeLa ^b^		Name	Genomic Coordinates ^e^	Accession No.		
**Antisense direction (lncRNA and miRNA)**								
^ hsa-mir-7704	2798 (100)	0	+	HAGLR	chr2:176173189-176188958	NR_033979.2	−	exon
hsa-mir-99b	366 (97.5)	25,823	+	SPACA6P-AS1	chr19:51685363-51693456	NR_108100.1	−	Splice junction
hsa-mir-125a	209 (99.5)	7587						exon
hsa-let-7e	532 (99.8)	5326						exon
hsa-mir-3615	46 (95.65)	646	+	SLC9A3R1-AS1	chr17:74747319-74748912	ENST00000585285.1	−	exon
^ hsa-let-7i	16,229 (99.75)	243,109	+	LINC01465	chr12:62601751-62603690	ENST00000408887.3	−	Part of exon (first 5 nt)
hsa-mir-196b	27 (100)	959	−	HOXA10-AS	chr7:27168899-27171915	NR_046609.1	+	exon
				HOXA10-HOXA9	chr7:27162438-27180261	NR_037940.1	-	intron
hsa-mir-29a	57 (89.47)	3818	−	LINC00513	chr7:130853732-130930680	ENST00000653887.1	+	intron
				LINC-PINT	chr7:130791264-131107928	ENST00000642963.1	−	intron/part of exon
^ hsa-mir-1246	3365 (100)	1025	−	LINC01117	chr2:176495255-176655967	ENST00000652995.1	+	intron
hsa-let-7d	285 (97.54)	5172	+	LINC02603	Isoform 1: chr9:94176569-94204566	NR_046163.1	−	intron
					Isoform 2: chr9:94176570-94204566	NR_046165.1		
					Isoform 3: chr9:94176569-94259311	NR_160773.1		
hsa-mir-196a-1	389 (98.71)	4663	−	HOXB-AS4	Isoform 1: chr17:48628698-48634932	NR_046611.1	+	intron
					Isoform 2: chr17:48629521-48634932	NR_170224.1		
hsa-mir-149	10 (100)	431	+	GPC1-AS1	chr2:240449379-240456700	NR_161169.1	−	intron
hsa-mir-10a	146 (100)	17,518	−	HOXB-AS3	chr17:48549630-48602332	ENST00000465846.6	+	intron
hsa-mir-15b	14 (85.71)	800	+	TRIM59-IFT80	Isoform 1: chr3:160227454-160449838	NR_148401.1	−	intron
hsa-mir-16-2	30 (80)	3123			Isoform 2: chr3:160256986-160449838	NR_148402.1		
					Isoform 3: chr3:160256986-160485747	NR_148403.1		
**Sense direction (lncRNA and miRNA)**								
hsa-mir-612	15 (0)	0	+	NEAT1	chr11:65422798-65445540	NR_131012.1	+	exon
hsa-mir-30c-2	192 (99.48)	19,157	−	LINC00472	Isoform 1: chr6:71343427-71420130	ENST00000651778.1	−	intron/exon
^ hsa-mir-30a	2311 (98.91)	79,239	−		Isoform 2: chr6:71373251-71420148	ENST00000625013.2		intron
hsa-mir-92b	235 (100)	4963	+	THBS3-AS1	Isoform 1: chr1:155194997-155205489	NR_183234.1	+	exon
					isoform 2: chr1:155194997-155205489	NR_183235.1		
					Isoform 3: chr1:155194997-155205489	NR_183236.1		
					Isoform 4: chr1:155194997-155205489	NR_183237.1		
					Isoform 5: chr1:155194997-155205489	NR_183238.1		
hsa-mir-143	12 (100)	704	+	CARMN	Isoform 1: chr5:149406845-149432836	NR_105059.1	+	exon
					Isoform 2: chr5:149406845-149432836	NR_105060.1		
hsa-mir-15a	13 (30.77)	20	−	DLEU2	Isoform 1: chr13:49982549-50125541	NR_152566.1	−	intron
					Isoform 2: chr13:50043436-50082041	NR_152567.1		
					Isoform 3: chr13:50043436-50082041	NR_152568.1		
hsa-mir-16-1	11 (100)	1196			Isoform 4: chr13:50049189-50082041	NR_152569.1		intron
					Isoform 5: chr13:50026695-50082041	NR_152571.1		
					Isoform 6: chr13:50049189-50082041	NR_152570.1		
					Isoform 7: chr13:50026695-50082041	NR_152572.1		
hsa-mir-421	15 (0)	207	−	FTX	chrX:74028136-74293574	NR_028379.1	−	intron
hsa-mir-374a	42 (85.71)	2070						intron
hsa-mir-374b	16 (93.75)	1941						intron
hsa-mir-4449	74 (0)	1	+	LINC01618	chr4:52,712,394-52,866,821	ENST00000650700.1	+	intron
				DANCR	Isoform 1: chr4:52712394-52714138	NR_024031.2		
					Isoform 2: chr4:52712394-52720697	NR_145129.1		
					Isoform 3: chr4:52712394-52720696	NR_145130.1		

^a^ SF, spliceosomal fractions of HeLa cells; ^b^ CE, cell extract of HeLa cells; ^c^ percentage mature miRNA; ^d^ excluding lncRNA which are miRNA host genes; and ^e^ GRCh38/hg38. ^ Indicates one of the top 20 abundant spliceosomal miRNAs in HeLa cells, according to Mahlab-Aviv et al., 2018 [18]. Table composed from data of ref. [18].

### 3.1. Antisense lncRNA and Spliceosomal miRNA Interaction in Breast and Cervical Cancer Cells

**Figure 1:**
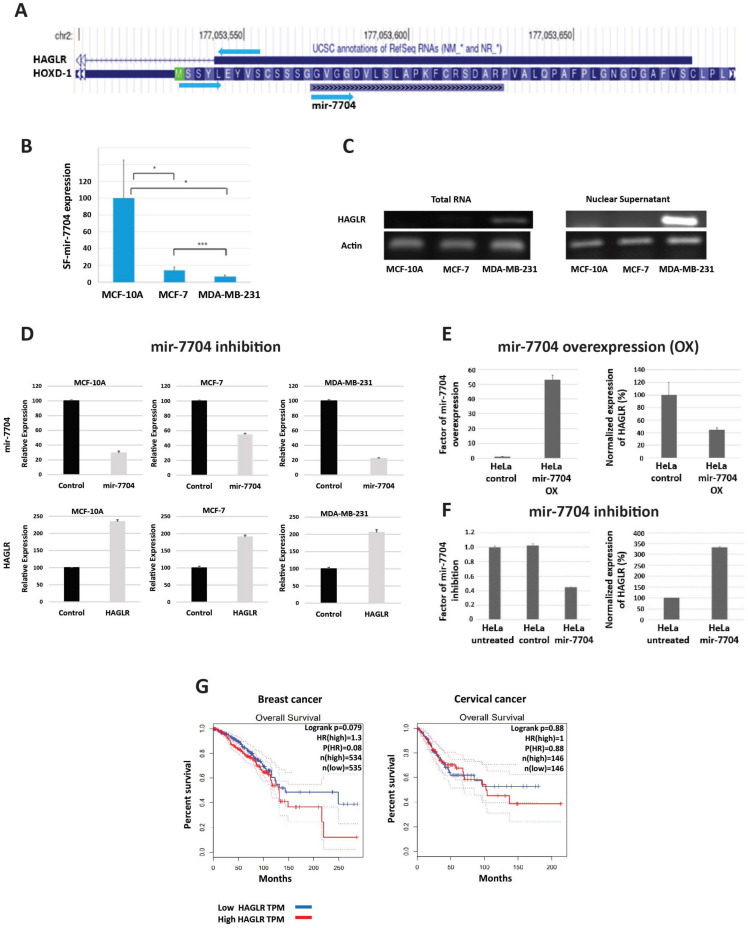
Negative regulation of HAGLR by spliceosomal mir-7704 in breast and cervical cancer cells. (**A**) UCSC Genome browser view indicates the overlap of mir-7704 with HOXD1 and the lncRNA HAGLR. (**B**) Average expression levels (RNA-Seq) and standard error of mir-7704 from the spliceosome fractions from MCF-10A, MCF-7, and MDA-MB-231 cells. Pair statistics are marked for <0.1 (*) and <0.001 (***). (**C**) Expression of HAGLR (RT-PCR) measured from total RNA and nuclear RNA in the three tested breast cell lines. (**D**) Inhibition of mir-7704 expression upregulates the expression of HAGLR. Results of quantitative real-time PCR (qRT-PCR) analysis of the effect of mir-7704 inhibition on the nuclear expression of HAGLR in MCF-10A, MCF-7, and MDA-MB-231 cells. Transfection with Anti-miR-7704 inhibitor resulted in the downregulation of mir-7704 (upper panel) and an increase in the expression level of HAGLR mRNA (lower panel) relative to the control (non-silencing anti-mir). HAGLR expression level was normalized to the internal control of ß-actin expressed from the same preparation. (**E**) Overexpression of mir-7704 leads to increase in the level of mir-7704 in HeLa cells, as measured by qRT-PCR. This increase in the level of mir-7704 is accompanied by significant decrease in the level of HAGLR mRNA compared to control (empty vector). (**F**) Downregulation of mir-7704 in HeLa cells leads to increase in the expression level of HAGLR mRNA compared to control (non-silencing anti-mir). The expression levels of HAGLR were normalized to ß-actin expression from the same preparation. (**G**) Survival curve analyses of HAGLR in breast and cervical cancer, based on data from GEPIA [41], The solid line represents the survival curve, and the dashed lines represent the 95% confidence interval. Data of (**B**–**D**) adapted from ref. [5], and (**E**,**F**), from ref. [18].

**Figure 2:**
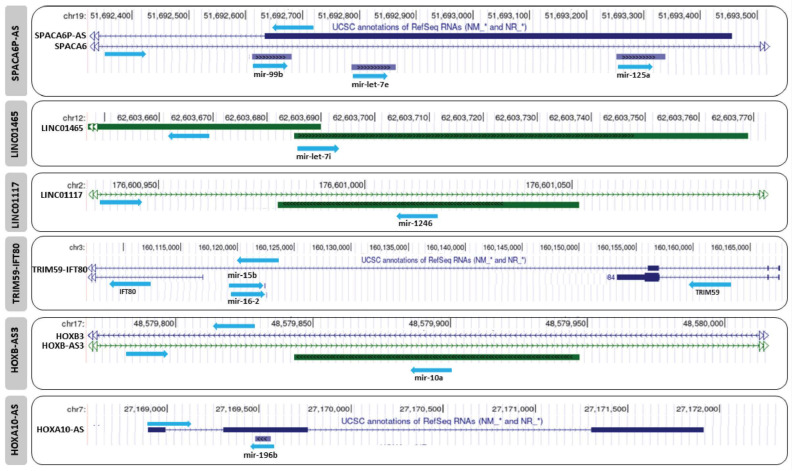
UCSC Genome browser view, indicating the overlap between the discussed miRNAs and their potential lncRNA crosstalk targets.

Here, we have chosen to focus on five lncRNAs: HAGLR; SPACA6P-AS (SPACA6 Antisense RNA 1); LINC01465 (Long Intergenic Non-Protein-Coding RNA 1465); LINC01117 (Long Intergenic Non-Protein-Coding RNA 1117); and TRIM59-IFT80. These lncRNAs are expressed in the antisense direction to seven miRNAs expressed in supraspliceosomes of both breast and cervical cancer cells and can thus be targets or involved in crosstalk with the respective miRNA (Figure 1 and Figure 2).

#### 3.1.1. HAGLR (HOXD Antisense Growth-Associated Long Non-Coding RNA)

HAGLR, known also as HOXD-AS1, is located between the HOXD1 and HOXD3 genes on chromosome 2q31.1 (Figure 1A) and has been reported to play a critical role in the development and progression of different human cancers, including bladder, cervical, colorectal, gastric, ovarian, prostate, glioma, hepatocellular carcinoma, melanoma, osteosarcoma, and non-small cell lung cancer [38]. HAGLR was also reported to promote the progression of triple-negative breast cancer (TNBC) by activation of the Wnt signaling pathway [39]. TNBC, one of the most aggressive breast cancer diseases, which lacks treatment, is characterized by a lack of estrogen receptor (ER), progesterone receptor (PR), and human epidermal growth factor receptor 2 (HER-2) [39]. HAGLR was found to be highly expressed in the cytoplasm of TNBC tissues and cells. Furthermore, inhibiting HAGLR suppressed cell proliferation, migration, and invasion and promoted cell apoptosis in TNBC [39]. 

HAGLR also has the ability to act as an miRNA decoy to promote cancer progression. An example is the role of HAGLR in modulating the miR-130a/E2F8 (E2F transcription factor 8) axis in glioma [33] and the promotion of neuron differentiation through the miR-130a-3p-MeCP2 axis [40]. HAGLR also plays a role in the miR-130a-3p/SOX4 (SRY-box 4) axis in liver cancer and in the miR-608/FZD4 (frizzled class receptor 4) axis in ovarian cancer. Furthermore, through the regulation of SOX4 by HAGLR, HOTAIR is activated and indirectly activates EZH2 and MMP2 (matrix metallopeptidase 2) to facilitate hepatocellular carcinoma metastasis [33]. HAGLR was also found to upregulate zinc finger E-box binding homeobox 1 (ZEB1) through binding with miR-130a-3p to promote resistance to cisplatin (chemotherapy medication used to treat several types of cancers) in cervical cancer [38].

Focusing on the crosstalk of HAGLR with spliceosomal miRNA, Mahlab Aviv et al. [5,18] noticed that has-mir-7704, which is found within the supraspliceosome of both breast and cervical cancer cells, is overlapping with HAGLR (NR_033979.2) and expressed in the opposite direction (Figure 1A), suggesting a crosstalk between HAGLR and mir-7704 [5,18]. When the expression of HAGLR and mir-7704 was analyzed in two breast-cancer-derived cell lines (MCF-7 and MDA-MB-231) and a non-tumorigenic cell line (MCF-10A), which are often used as cellular models for breast cancer, negative correlation was found between the expression of HAGLR and mir-7704 [5]. Namely, the expression of mir-7704 in the supraspliceosome is significantly much higher in normal cells (MCF-10A) compared to breast cancer cells (MCF-7 and MDA-MB-231) (Figure 1B), while the level of HAGLR increases in breast cancer cells (Figure 1C). Importantly, inhibition of mir-7704 caused an increase in HAGLR expression in breast cancer cells, demonstrating negative regulation of the oncogenic lncRNA HAGLR by spliceosomal mir-7704 (Figure 1D). Furthermore, increasing mir-7704 levels attenuated the MDA-MB-231 cell division rate [5]. These findings suggest that HAGLR, which is expressed at high levels in breast cancer cells, could serve as a target for the diagnosis and treatment of breast cancer.

Interestingly, analysis of the relative levels of mir-7704 found in the spliceosome fractions of non-tumorigenic relative to cancer-derived breast cell lines revealed that the level of spliceosomal mir-7704 decreased with malignancy. This trend is different from that reported in the literature and from the observations from large cohorts of breast cancer patients, in which mir-7704 increased with malignancy [5]. These findings suggest that spliceosomal mir-7704 acts on nuclear target/s which are different from the cytoplasmic ones. Indeed, the above studies [5] identified HAGLR as the nuclear target of mir-7704.

mir-7704 is also highly expressed (2.01% of spliceosomal miRNA) in supraspliceosomes of HeLa cells—cervical cancer cells [18]. Real-time PCR analysis of HeLa cells overexpressing mir-7704 (Figure 1E) revealed significant reduction in the level of HAGLR. On the other hand, analysis of cells that were transfected with anti-mir-7704 inhibitor showed that downregulation of mir-7704 resulted in upregulation in the level of HAGLR (Figure 1F) [18]. It can thus be further concluded that mir-7704 negatively regulates the expression of the oncogenic lncRNA HAGLR [18].

The findings that HAGLR is expressed at high levels in cervical cancer cells compared to control [38,42], that knockdown of HAGLR significantly inhibited cervical cancer cell proliferation and colony formation, and that cervical cancer cell growth can be suppressed by inactivating the Ras/ERK pathway [38,42] reinforce our conclusion of the importance of HAGLR as a target for breast and cervical cancer. 

Further support for our results and hypothesis comes from survival curve analysis of HAGLR in breast and cervical cancer based on gene expression levels [41], showing that low levels of HAGLR are associated with patients’ survival (Figure 1G).

To conclude, HAGLR regulates the growth, invasion, and migration of tumor cells through different underlying mechanisms in different types of cancer, such as breast [5,39] cervical, lung, glioma, gastric, melanoma, ovarian, prostate, and more [31,33,38]. Thus, it can be considered as a promising diagnostic/prognostic biomarker or a novel therapeutic target for cancers. Modified antisense oligonucleotides have been used successfully to affect gene expression in research and medicine [43,44,45]. We thus propose the use of modified oligos, which mimic the effect of mir-7704 to suppress the expression of HAGLR in breast and cervical cancer. Here, we highlight a new player, namely spliceosomal mir-7704, and the crosstalk between HAGLR and spliceosomal mir-7704 as a novel target for the diagnosis and therapeutics of breast and cervical cancer. 

#### 3.1.2. SPACA6P-AS (SPACA6 Antisense RNA 1)

SPACA6P-AS, also known as LINC01129, is antisense to the SPACA6 gene, located on chromosome 19q13.41. SPACA6P-AS also overlaps and is antisense to an miRNA cluster (miRNA- 99b, let-7e, and miRNA-125a) (Figure 2). Analysis of the expression levels of this miRNA cluster in the supraspliceosome of breast cancer cells compared to normal breast cells (MCF-7 and MDA-MB-231 compared to MCF-10A normal breast cells) revealed changes in expression level in breast cancer (Table 1). Importantly, the genomic location of mir-99b-5p is antisense to the splice junction of exon 1 and intron 1 of the lncRNA SPACA6P-AS (NR_108100.1) [18]. Thus, miR-99b-5p is fully complementary to the 5′ splice junction of intron 1 of SPACA6P-AS, spanning from positions −6 to +16 (22 nt) with respect to the splice junction [18]. RNA-Seq analysis revealed that miR-99b sequences in the supraspliceosomes of breast cancer cells are those of mir-99b-5p (100% in MDA-MB-231 and over 94% in MCF-10a and MCF-7 cells, Table 1). This complementarity suggests that spliceosomal mir-99b can affect the splicing of SPACA6P-AS by competing with the binding of U1 and U6 snRNPs, which is required for the splicing of this lncRNA. A crosstalk between spliceosomal mir-99b and SPACA6P-AS, affecting the expression level of mir-99b and the splicing of this lncRNA, was proposed [18], with mir-99b playing a role in determining the ratio between spliced and un-spliced LINC01129 RNA. Supraspliceosomal mir-99b might also play a role in the network of SPACA6 and LINC01129 [18]. As the expression level of mir-99b is highest in the metastatic MDA-MB-231 cells, it is anticipated that the level of un-spliced SPACA6P-AS is highest in these malignant cells compared to normal breast cells. However, since the expression level of spliceosomal let-7e and mir-125a is high in normal breast cells compared to breast cancer cells (Table 1), these latter spliceosomal miRNAs likely repress the expression of SPACA6P-AS, while mir-99b affects the ratio of spliced and un-spliced SPACA6P-AS. Little is known about the functional role of SPACA6P-AS and nothing about the different roles of spliced and un-spliced SPACA6P-AS. However, it should be noted that SPACA6P-AS is considered as a risk lncRNA, which is inversely related to breast cancer survival [46]. 

This miRNA cluster is also expressed in supraspliceosomes of cervical cancer cells, but the expression of this miRNA cluster in the supraspliceosome is lower than in the cytoplasm (Table 2) [18].

A recent study showed that SPACA6P-AS form a ceRNA regulatory network, involving mir-125a and its mRNA targets (Lin28b, MMP11, SIRT7, and Zbtb7a) in hepatocarcinoma cells, in which mir-125a can regulate the expression of SPACA6P-AS and vice versa. Overexpression of SPACA6P-AS regulates onco-suppressive mir-125a, resulting in upregulation of oncogenic mir-125a targets [47].

#### 3.1.3. LINC01465 (Long Intergenic Non-Protein-Coding RNA 1465)

LINC01465 is located on chromosome 12q14.1 and is suggested to have a potential role in cell migration of epithelial ovarian cancer (EOC) cells [48]. The sequence of mir-let-7i is complementary to the beginning of the exon sequences of an isoform (ENST00000408887.3) of this lncRNA (Figure 2). 

Analysis of the expression levels of this miRNA in the supraspliceosomes of breast cancer cells compared to normal breast cells showed a high level in breast cancer cells compared to control (Table 1) [5].

In addition, based on our RNA-seq analysis in HeLa cells [18], mir-let-7i is relatively highly expressed in the spliceosome fractions of these cells (Table 2). It is the second-most highly expressed spliceosomal miRNA in HeLa supraspliceosomes, occupying 11.76% of all the supraspliceosomal miRNAs. Although experiments are required to determine the effect of LINC01465 on breast and cervical cancer, we suggest that spliceosomal mir-let-7i might inhibit the expression of LINC01465 (ENST00000408887.3) isoform. mir-let-7i is expressed also in an antisense direction to the promotor region of the isoform NR_121682.1 of LINC01465 and thus could potentially affect the expression of this isoform as well. We, therefore, highlight the LINC01465:spliceosomal-mir-let-7i interaction as a potential novel target for the diagnosis and treatment of breast and cervical cancer.

#### 3.1.4. LINC01117 (Long Intergenic Non-Protein-Coding RNA 1117)

LINC01117 is located on chromosome 2q31.1. Little is known about this lncRNA, yet it was found upregulated in breast cancer compared to normal tissues [49]. Also, its expression level is found elevated in breast cancer compared to normal tissues, according to GEPIA database [41]. LINC01117 is antisense to miRNA-1246 (Figure 2), which is expressed at significant high levels in the supraspliceosome of normal breast cells compared to breast cancer cells [5]. Because miRNA-1246 overlaps an intron of LINC01117 (ENST00000652995.1), it is hard to predict how the decreased level of spliceosomal mir-1246 in breast cancer cells [5] might affect the level of LINC01117 in breast cancer compared to normal cells, and further studies are required to resolve that. It should be noted that the decrease in the level of spliceosomal mir-1246 with malignancy (Table 1) is different from that reported from a large cohort of breast cancer patients, in which mir-1246 acts as oncogenic [5].

It should be pointed out that mir-1246 is highly expressed in supraspliceosomes of cervical cancer cells (2.42% of spliceosomal miRNA) and that the expression of mir-1246 in the supraspliceosome is higher than in the cytoplasm, suggesting both a nuclear and cytoplasmic role for mir-1246 in HeLa cells (Table 2) [18]. 

#### 3.1.5. TRIM59-IFT80: Located on Chromosome 3q25.33

This lncRNA is a merged product of the genes IFT80 and TRIM59 (Figure 2). It is expressed antisense to mir-15b and mir-16-2, which are complementary to intron sequences of TRIM59-IFT80 (NR_148401.1/NR_148402.1/NR_148403.1). Analysis of mir-16b expression in the supraspliceosomes of breast cancer cells [5] revealed that the expression level of mir-16-2 is higher in breast cancer cells (MDA-MB-231) compared to the normal breast cells (MCF-10A) (Table 1). It is thus possible that the level of TRIM59-IFT80 will be affected more in breast cancer cells, although it is not clear if and how the complementarity to intronic sequences might affect the splicing of TRIM59-IFT80 and thus its level. Experiments are required to determine the effect. mir-15b and mir-16-2 are also expressed in the supraspliceosomes of cervical cancer cells; however, their expression is high in the cytoplasm compared to the spliceosome fractions, suggesting a cytoplasmic role for these miRNAs in HeLa cells (Table 2) [18].

### 3.2. Antisense lncRNA and Spliceosomal miRNA Interaction in Cervical Cancer Cells

In addition to the list of miRNAs antisense to an lncRNA, which are expressed in the supraspliceosomes of both breast and cervical cancer cells (Table 1 and Table 2), we highlight here HOXB-AS3 (HOXB Cluster Antisense RNA 3) and HOXA10-AS (HOXA Cluster Antisense RNA 4), which are found in supraspliceosomes of the cervical cancer cells (but not in breast cancer cells) and might affect lncRNA expression there [18]. HOXB-AS3 and HOXA10-AS are also known to be part of a ceRNA network [50,51]. 

#### 3.2.1. HOXB-AS3 (HOXB Cluster Antisense RNA 3)

HOXB-AS3 is located on chromosome 17q21.32. Due to alternative splicing, HOXB-AS3 lncRNA can generate a number of splice isoforms. One isoform, NR_033201.2, encodes a 53 aa long micropeptide. In normal colon cells, this micropeptide directly interacts with hnRNPA1 to affect pyruvate kinase gene (PKM) splicing, generating an mRNA isoform including exon 9 (but not 10) encoding PKM1. PKM1 is important for suppressing cellular aerobic glycolysis and tumorigenesis. On the other hand, in colon cancer, the micropeptide is not expressed and exon 10 is included (rather than exon 9), generating mRNA isoform encoding for PKM2, which stimulates tumor growth [52]. Another HOXB-AS3 isoform (NR_033202.2), which does not encode for the micropeptide, can promote the proliferation of cancer cells (acute myeloid leukemia) by enhancing the transcription of ribosomal RNAs [53].

HOXB-AS3 is also known as part of a ceRNA network in ovarian cancer (OC). It is upregulated in OC and correlated with the prognosis of OC patients [50]. Furthermore, when HOXB-AS3 acts as a sponge of mir-378a-3p, this may lead to upregulation of LDHA, which is a target of mir-378a-3p, to modulate the Warburg effect in OC cells. Moreover, the HOXB-AS3/mir-378a-3p/LDHA axis may enhance OC growth, invasion, and migration and thus it could serve as an independent prognostic marker for OC patients [50]. Thus, HOXB-AS3 can act as a tumor suppressor or an oncogenic lncRNA depending on its alternative splicing [54].

RNA-Seq analysis of spliceosomal miRNA in HeLa cells [18] revealed that mir-10a, which is antisense to intronic sequences of lncRNA HOXB-AS3 (ENST00000465846.6) (Figure 2), is found in supraspliceosomes of HeLa cells. It has a higher expression level in the cytoplasm compared to the supraspliceosome fractions (Table 2). It is possible that mir-10a plays a role in both the spliceosome and the cytoplasm. However, experimental data are required to determine if this lncRNA could be a target to spliceosomal mir-10a.

#### 3.2.2. HOXA10-AS (HOXA Cluster Antisense RNA 4)

HOXA10-AS is located on chromosome 7p15.2, and mir-196b is located in the antisense direction to exon 2 of HOXA10-AS (NR_046609.1) (Figure 2). It has been reported that HOXA10-AS is involved in several cancers. For example, the HOXA10-AS/mir-340-3p/HTR1D ceRNA axis promotes the malignant progression of pancreatic cancer via the PI3K-AKT signaling pathway [51]. Decrease in the level of lncRNA HOXA10-AS reduced the sponging of mir-340-3p, resulting in an increase in mir-340-3p and a subsequent decrease in HTR1D to ultimately suppress the malignancy of cancer [51]. Also, HOXA10-AS is highly expressed in nasopharyngeal carcinoma (NPC) cells [55]. It was demonstrated that HOXA10-AS, activated by E2F1, leads to an increase in the proliferation and migration of NPC cells, through the sponging of mir-582-3p. This sponging of mir-582-3p leads to upregulation of its target gene Ras-related protein Rab-31 (RAB31) and facilitates cell proliferation and migration of NPC cells [55].

In principle, mir-196b, which is complementary to exon 2 of HOXA10-AS, could affect the splicing of HOXA10-AS and thus its expression. Our RNA-seq analysis of miRNA in the supraspliceosomes of HeLa cells shows a low expression level (Table 2) of this miRNA [18]. Further experiments are needed to determine the effect of spliceosomal mir-196b on cervical cancer. Nonetheless, we point out mir-196b as a potential candidate for affecting the expression of HOXA10-AS in those cancers where mir-196b is expressed in the supraspliceosome.

### 3.3. Sense Spliceosomal miRNA and lncRNA Crosstalk in Breast and Cervical Cancer Cells 

Table 1 and Table 2 also include lncRNAs, known to be involved in cancer, which are expressed from the same strand as an miRNA that is found in the supraspliceosome. These cases, which likely portray additional cases of lncRNA:miRNA crosstalk that could be relevant to cancer, will not be elaborated here. We only comment briefly on three cases of abundant or cancer-documented cases. 

In brief, an example is the case of NEAT1 lncRNA, which is essential for the assembly of nuclear paraspeckles [56]. NEAT1 was demonstrated to have a role in tumor initiation, growth, and metastasis in breast cancer [57]. Notably, mir-612, which is expressed from the same region of the 3′ end of the long isoform of NEAT1 and in the same direction, is one of the most abundant spliceosomal miRNAs in the MCF-10A normal breast cell line (Table 1), but is not found among the spliceosomal miRNAs of the breast cancer cells [5] (Table 1). The sequences of spliceosomal mir-612 in MCF-10A cells are not limited to mature miRNAs and mainly represent overlapping regions. As the 3′ end of the long isoform of NEAT1 is involved in the stability of this lncRNA, which forms paraspeckles [58], a potential crosstalk with spliceosomal mir-612 might affect the expression of NEAT1. Notably, analysis of the changes in the level of spliceosomal mir-612 of non-tumorigenic relative to cancer-derived breast cell lines revealed that spliceosomal mir-612 decreases with cancer. This trend is different from that reported from a large cohort of breast cancer patients, in which mir-612 acts as oncogenic [5]. It should be noted that the level of spliceosomal miR-612 is very low also in cervical cancer cells (Table 2) [18].

Another example of a cancer-relevant lncRNA is PVT1, known to be involved in cancer, which is significantly upregulated in breast cancer cell lines and promotes breast cancer cell proliferation and metastasis, through binding to mir-128-3p and UPF1 [59]. mir-1204, which is expressed in the same direction of an intron of PVT1, is found at low levels in the spliceosomal miRNAs of MCF-10A cells (Table 1) [5].

An additional case is LINC00472, known as a biomarker for cancer, and due to its lower expression in tumors, it could act as a tumor suppressor in breast cancer [60] and renal cell carcinoma [61]. Furthermore, LINC00472 inhibits oral squamous cell carcinoma progression via the mir-4311/GNG7 axis [62]. Based on the RNA-seq analysis in breast cancer cells [5], the expression level of spliceosomal mir-30a, which is expressed in the same direction of an intron of LINC00472, increases significantly in metastatic MDA-MB-231 cells compared to non-malignant MCF-10A cells, acting as an oncomir. This is different from that reported in the literature and from the observations from large cohorts of breast cancer patients, in which mir-30a acts as a tumor suppressor [5], suggesting nuclear targets. Although it is hard to predict the effect of spliceosomal mir-30a on the expression of LINC00472, an optional effect is through sequestering factors that bind to LINC00472. A similar trend is found in ovarian cancer cells, where mir-30a is highly expressed in the supraspliceosome (Table 2) and is one of the top 20 HeLa spliceosomal miRNAs [18].

It should be noted that in cases where an lncRNA and a spliceosomal miRNA are expressed from the same genomic region, in the same direction, a possible effect is by sequestering factors from each other, dependent on which of them is more abundant in the nucleus. However, the power of prediction in these cases is limited, and thus future experimental data are required. 

## 4. Conclusions

**Figure 3:**
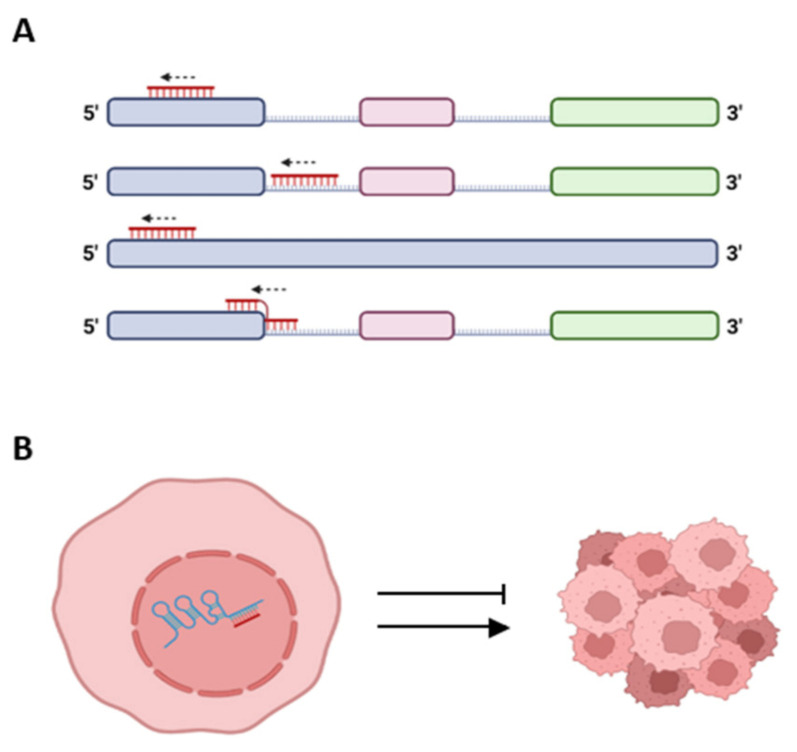
Proposed types of crosstalk between lncRNA and respective spliceosomal miRNA, schematic drawing. (**A**) Complementarity between spliceosomal miRNA (red) and different regions along the sequence of an lncRNA (pastel colors). lncRNA colored blocks—exons; and lines—introns. (**B**) A scheme portraying potential interactions through base pairing between lncRNA (blue) and spliceosomal miRNA (red) in the nucleus, which can lead to promotion or inhibition of cancer. Created with BioRender.com (accessed on 17 June 2023).

Numerous regulatory effects of lncRNAs on gene expression have been elucidated through their interaction with DNA, RNA, proteins, and combinations of them. These interactions included trans and cis interactions. Several cases of cis-acting lncRNAs which activate or repress other genes via interaction with the DNA of promoters or enhancers when complexed with proteins have been studied [31]. The regulatory function/s of lncRNAs may also vary with alternative splicing, generating tumorigenic or tumor suppressor splice variants from the same lncRNA [54]. The finding of miRNA expressed in supraspliceosomes [2,4,5,18] opened up new candidates for cis-acting targets on lncRNA and vice versa. Here, we highlight potential cases of crosstalk between lncRNA, involved in breast and cervical cancer, and supraspliceosomal miRNA expressed from the same genomic region. Specifically, we highlight those miRNAs that complement lncRNA sequences and especially those that complement exonic or splice junction sequences of an lncRNA and thus, through RNA:RNA base pairing, are likely to affect the expression of an lncRNA involved in cancer (Figure 3). 

Importantly, four of the discussed spliceosomal miRNAs (mir-7704, mir-1246, mir-612, and mir-30a) show inverse directionality when comparing the changes in their expression level in non-malignant (MCF-10A) compared to metastatic (MDA-MB-231) breast cancer cells to data from large cohorts of breast cancer patients [5]. The expression levels of spliceosomal mir-7704, mir-1246, and mir-612 decreased with increased carcinogenesis, while the clinical data indicate them as oncogenic. In the case of spliceosomal mir-30a, the expression level increased with carcinogenesis, while the clinical data are consistent with mir-30a being a tumor suppressor. These contradictions between the expression trend of these spliceosomal miRNA and the clinical outcome indicate that these miRNAs are acting in the nucleus on different targets than those in the cytoplasm [5]. Therefore, the lncRNAs discussed here, which are expressed from the same genomic region, are novel potential candidate targets. A proven example is the case of the lncRNA HAGLR and mir-7704, which are expressed in the opposite direction from the same genomic region. It was demonstrated that mir-7704 negatively regulates the expression of the oncogenic HAGLR in breast [5] and cervical cancer cells [18]. 

RNA:RNA base pairing is a major player in gene regulation and specifically in the spliceosome, where base pairing of spliceosomal U snRNAs with pre-mRNA sequences plays a crucial role in splicing [8,9,10,11,12]. An additional example of a functional RNA:RNA base pairing within the spliceosome is the base pairing of spliceosomal SNORD27 with E2F7 pre-mRNA, affecting alternative splicing [17]. Furthermore, modified antisense oligonucleotides have been used successfully to affect splicing and gene expression in research and medicine [43,44,45]. The current experimental data supporting a crosstalk between spliceosomal miRNA and lncRNA expressed from the same genomic location in the cases of HAGLR [5,18] and SPACA6P-AS [47] are in support of the proposal for a crosstalk in additional examples, and highlight the potential implications of such interactions on discovering novel targets for cancer therapeutics. 

Thus, through RNA:RNA base pairing and changes in the crosstalk through changes in the level of one of the partners (either miRNA or lncRNA), as occur in cancer, an effect on the expression of the second component could occur, generating changes that might be relevant to breast and cervical cancer. 

Because the expression of spliceosomal miRNA is cell-type-specific, it is anticipated that the list of cis-interacting lncRNA:spliceosomal miRNA presented here is just the tip of the iceberg. Furthermore, it is likely that such interactions are not limited to breast and cervical cancer cells and are likely relevant to additional types of cancer, where the relevant spliceosomal miRNAs are present. Future studies of spliceosomal miRNA in additional cancer cell types will resolve that. These interactions suggest novel targets for cancer therapeutics, through the use of modified oligos that could mimic the effect of the relevant miRNA. Furthermore, the unique and dynamic changes in expression of spliceosomal miRNA and lncRNA in different cancer cell types can be used as biomarkers for additional types of cancers and several diseases. These findings highlight the crosstalk of lncRNA with spliceosomal miRNA as a potential source for novel targets for cancer diagnosis and therapies.
